# GRANDPARENT CAREGIVERS: THE RELATION BETWEEN SOCIAL NETWORKS AND RESILIENCE

**DOI:** 10.1093/geroni/igz038.2507

**Published:** 2019-11-08

**Authors:** Nancy Mendoza, Christine Fruhauf

**Affiliations:** 1 The Ohio State University, Columbus, Ohio, United States; 2 Colorado State University, Fort Collins, Colorado, United States

## Abstract

Grandparents raising grandchildren experience multiple challenges as they take on the unexpected role of caring for their grandchildren, which usually occurs under stressful and stigmatizing conditions. Many of the challenges grandparents experience are well documented in the research. Less attention is given to understanding how a grandparent caregiver’s social network changes when s/he becomes a caregiver and how her/his social network influences resilience. Thus, the purpose of this study was to use social network analysis (SNA) to examine the relation between social networks and resilience in grandparents raising their grandchildren. This was done by conducting face-to-face interviews with twenty grandparents raising grandchildren after they completed a survey measuring social support, social isolation, and resilience. The interview protocol included questions related to participants’ social network, social support, and services. Prior to the interviews, using data from the surveys participants were identified as representing one of four resilience quadrants: resilient, maladaptive, competent, and vulnerable. Qualitative analysis of grandparent’s social networks across groups indicated resilient grandparent caregivers’ networks were structured in a way that provided more opportunities for the inflow of new information and resources. Whereas the proportion of professionals in maladaptive grandparent caregivers’ networks tended to be less than for other networks. This could suggest that for grandparent caregivers, having professionals in one’s network can be beneficial. Findings from the current study provide opportunities for future research such as identifying ways to help grandparent caregivers structure their social networks to promote resilience.

